# Vitally important – does early innate immunity predict recruitment and adult innate immunity?

**DOI:** 10.1002/ece3.1939

**Published:** 2016-02-19

**Authors:** Anke Vermeulen, Wendt Müller, Marcel Eens

**Affiliations:** ^1^ Department of Biology – Behavioural Ecology and Ecophysiology GroupUniversity of AntwerpUniversiteitsplein 1Wilrijk2610AntwerpBelgium

**Keywords:** Complement system, consistency in immune responses, haptoglobin, natural antibodies, nitric oxide, recruitment

## Abstract

The immune system is one of the most important adaptations that has evolved to protect animals from a wide range of pathogens they encounter from early life onwards. During the early developmental period this is particularly true for the innate immunity, as other components of the immune system are, as yet, poorly developed. But innate immunity may not only be crucial for early life survival, but may also have long‐lasting effects, for example if early life immunity reflects the functioning of the immune system as a whole. For this reason, we investigated the importance of four constitutive innate immune parameters (natural antibodies, complement activity, concentrations of haptoglobin, and concentrations of nitric oxide) for recruitment in free‐living great tits. We compared nestling immunity of recruits with nestling immunity of their nonrecruited siblings. We also investigated within individual consistency of these innate immune parameters for those individuals that recruited, which may be taken as a measure of immune capacity. In accordance with previous studies, we found a clear effect of tarsus length and a trend for body mass on the likelihood to recruit. Nevertheless, we found no evidence that higher levels of constitutive innate immunity as a nestling facilitated local recruitment. Furthermore, individual innate immunity was not consistent across life stages, that is to say, nestling immune parameters did not determine, or respectively, reflect adult innate immune parameters. This plasticity in innate immune components may explain why we did not find long‐lasting survival benefits.

## Introduction

The immune system is a unique defence system that provides animals with the ability to fight off invading pathogens and infections. It can be divided into two main types, innate immunity and adaptive immunity. Innate immunity is nonspecific and responds immediately, thus providing initial protection against infections. It consists of physical and chemical barriers, blood proteins and phagocytic cells (Abbas and Lichtman 2010). Adaptive immunity typically requires activation and provides the second line of defense (Grogan et al. 2008). However, it provides specific protection, since adaptive immunity involves lymphocytes and antibodies that express receptors that specifically recognize different antigens (Abbas and Lichtman 2010). Thus, the immune system is probably the most important adaptation to protect individuals against pathogens (Sheldon and Verhulst 1996). Indeed, individuals with stronger immune responses have higher survival probabilities (Saino et al. 1997; Christe et al. 1998; Soler et al. 1999; Møller and Saino 2004).

In birds, survival probabilities fluctuate throughout life, with mortality being particularly high during the postfledging period (e.g., due to predation, food competition, …)(Dhondt 1979; Naef‐Daenzer et al. 1999; Payevsky 2006). As a consequence only a small number of individuals manage to survive and recruit in the local breeding population (Dhondt 1979; Tinbergen and Boerlijst 1990; Naef‐Daenzer et al. 2001). Two well‐known predictors of postfledging survival are hatching date and fledging mass. Thus, birds fledging early in the season and birds with higher mass or size at fledging have higher survival chances (e.g., Both et al. 1999; Monrós et al. 2002; Radersma et al. 2015). However, physiological parameters such as telomere shortening, resistance to oxidative stress and prefledging oxidative damage have recently also been tested as potential predictors of local recruitment (Noguera et al. 2012; Losdat et al. 2013; Boonekamp et al. 2014). Besides these parameters, immunity probably also plays a crucial role as it provides protection from early life onwards (Klasing and Leshchinsky 1999; Levy and Netea 2014). On the one hand it affects the probability that an individual will survive during the nestling period (Hõrak et al. 1999). On the other hand, it has also been shown that nestling cell‐mediated immunity has a significant effect on the probability of local recruitment in blue tits (*Cyanistes caeruleus*) and spotless starlings (*Sturnus unicolor*) (Cichon and Dubiec 2005; Lopez‐Rull et al. 2011). In addition, it has been shown that hematological variables such as lymphocyte and heterophil counts can also be good predictors of recruitment (Lobato et al. 2005).

Innate immunity plays a particularly key role early in life, since young animals have very poor adaptive immune responses (Klasing and Leshchinsky 1999; Grindstaff et al. 2006). They are, therefore, much more dependent on their innate immune system and the maternal antibodies they received until the time that they themselves develop enough endogenous antibody levels (Klasing and Leshchinsky 1999; Grindstaff et al. 2006; Palacios et al. 2009; Niewiesk 2014). It is, therefore, plausible that innate immunity forms a foundation for early survival and that it provides a mechanism as to why early life immunity renders a survival benefit in the long‐term (Cichon and Dubiec 2005; Lopez‐Rull et al. 2011). Nestling innate immunity may already reflect the functioning of the innate immune system as a whole and respectively it may reflect the intrinsic capacities of the immune system. Furthermore, there are also heritable components of immunity (e.g., Cichon et al. 2006) and these may lead to consistent levels of immunity within an individual (Burleson et al. 2002; Yang et al. 2009; Ardia et al. 2011; Hegemann et al. 2013). Immunity is, of course, likely to be plastic and reflect environmental conditions (Ardia et al. 2011; see also Lucas and French 2012). Furthermore, immunity also passes through a developmental process that continues until adult levels are reached (Palacios et al. 2009; Stambaugh et al. 2011; Ygberg and Nilsson 2012).

In this study, we investigated whether constitutive innate immunity (=baseline immunity) is a foundation for local recruitment and adult immunity. We hypothesize that variation in innate immune parameters can be used as a predictor of local recruitment and that there is a positive relationship (consistency) between nestling immunity and adult immunity at the individual level.

## Materials and Methods

### Study sites and data sampling

**Figure 1:**
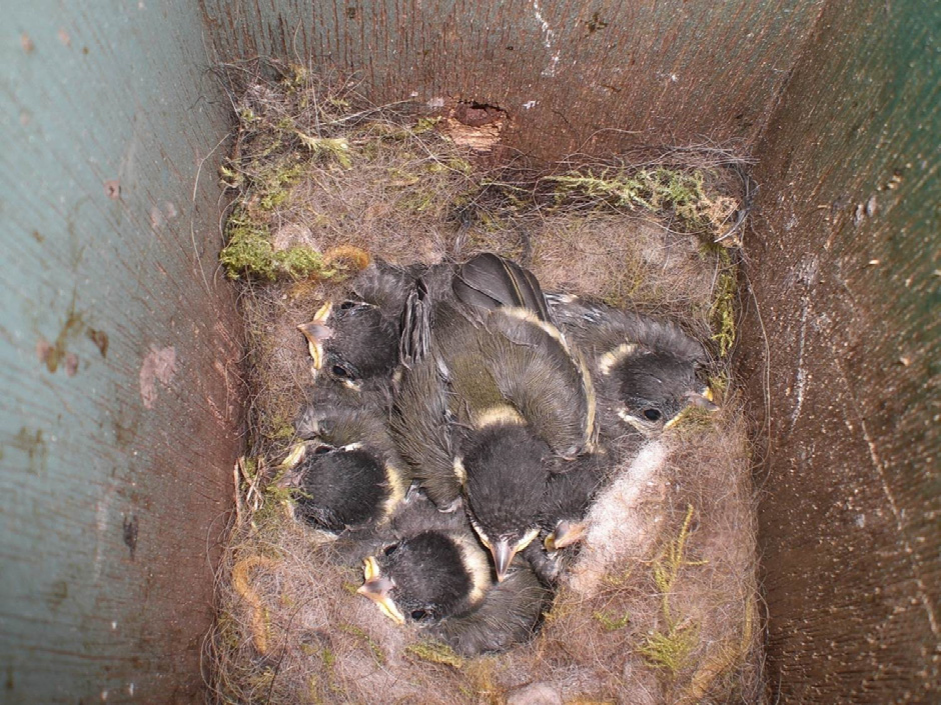
Top view of great tit nestlings in their nest box, almost ready to fledge.

The first part of this study (nestling immunity) was performed during the breeding season (April–May) of 2012, the second part (recruitment and adult immunity) during the following winter (2012–2013) and following breeding season (2013) in five well‐established great tit (*Parus major*) populations in and around Antwerp, Belgium (Hoboken: N 51 10 10, E 4 20 44.4 and N 51 9 48.3, E 4 20 48.9; Wilrijk: N 51 9 56.3, E 4 22 36.7 and N 51 9 47.2, E 4 24 10.2; Mortsel: N 51 10 28.2, E 4 27 37.3). All study sites consist of deciduous park areas and each site has approximately 30–116 great tit nest boxes (Fig. 1).

Nestling immunity: During the breeding season of 2012, we checked all nest boxes every other day to determine laying date, clutch size, start of incubation and the exact hatch day. When the nestlings were 14 days old, we collected a blood sample (±150 *μ*L) from the brachial vein using a Microvette CB 300 lithium‐heparin tube (Sarstedt), we provided the birds with a ring, and measured their body mass (precision 0.1 g) as well as their tarsus length (precision 0.01 mm). In total, we sampled 986 individual nestlings belonging to 146 nests. All blood samples were collected immediately after taking the birds out of the nest, to minimize the potential effects of stress on baseline immune parameters (Matson et al. 2006; Millet et al. 2007; Buehler et al. 2008). Blood samples were stored under cool conditions during transport and were centrifuged at 3293 g for 10 min when back in the lab. Resulting plasma samples were stored at −80°C until the start of the immunological assays.

Recruitment and adult immunity: during the following winter (November 2012 to February 2013), we conducted night checks (great tits sleep in the nest boxes during the winter) in all study areas in order to retrieve nestlings that had established a territory in one of our study locations. Recruitment is here defined as establishing a territory either in winter or during the breeding season. Based on the metal ring numbers, we were able to identify 17 birds that were born in our study area. For all of these recruits blood was sampled and measurements were taken in a similar way as described above and then they were subsequently returned to their nest box. During the breeding season of 2013 (April–May) we continued the search for recruits from the 2012 breeding season by catching birds on the nest or via live traps at the entrance hole while they were feeding nestlings of 8–15 days old. In this way we identified 19 additional recruits for which we collected a blood sample and measurements in a similar way as described above. Since in general, recruits are considered to be birds that enter the breeding population (Nicolaus et al. 2008), we additionally analyzed data of only birds that eventually bred.

We measured natural antibodies (NAbs) and complement activity, which are two interrelated noncellular components of innate immunity. NAbs broadly recognize and bind to antigens which can result in activation of the complements cascade and ends with the lysis of foreign cells (Boes 2000; Ochsenbein and Zinkernagel 2000; Matson et al. 2005; Murphy et al. 2012). Furthermore, we measured the concentrations of the acute phase protein (APP) haptoglobin (Hp). These APPs are typically synthesized by hepatocytes in response to cytokines released by macrophages when bacteria are present (Owen‐Ashley and Wingfield 2007; Cray et al. 2009; Coon et al. 2011; Murphy et al. 2012) and they have several antimicrobial functions, such as opsonizing bacteria and activating the complement cascade (Murphy et al. 2012). Concentrations are known to rise significantly in response to an acute infection, trauma, or inflammation (Murata et al. 2004; Quaye 2008; Cray et al. 2009; Matson et al. 2012). As a last innate immune parameter, we measured nitric oxide (NO) which is a multifunctional signaling molecule and acts as a vasodilator, neurotransmitter and a modulator of inflammatory processes. Assessing nitric oxide can provide useful information on individual variation in work load, physiological condition, and health state (Bourgeon et al. 2007; Sild and Horak 2009).

For this particular study, we initially sampled a total of 986 nestlings belonging to 146 different nests. Immunological assays were conducted for samples of nestlings that recruited (*N* = 36) and for samples of their nonrecruited siblings (*N* = 119) belonging to the same nest (*N* = 26). We also analysed the blood samples of the adult birds that were found as recruits (*N* = 17 during the following winter, *N* = 19 during the breeding season, *N*_total_ = 36, *N*_nests_ = 26). The analysis to test whether there were differences in body weight or tarsus length between birds who recruited and birds who failed to recruit was based on the previous mentioned data (recruits and their nonrecruited siblings) plus some randomly analyzed samples from nests containing no recruits (*N*_total_ = 447).

### Immunological assays

#### Hemolysis–hemagglutination assay

Levels of natural antibodies and complement activity present in the blood were assessed using the hemolysis–hemagglutination assay as developed by Matson et al. (2005) with some minor alterations (Vermeulen et al. 2015). Briefly, we treated all plates with a blocking solution consisting of milk powder and Dulbecco's phosphate buffered saline (PBS) and subsequently washed them three times using a PBS‐TWEEN 20 solution. The serial dilution (1:2) was based on 15 *μ*L of plasma and 15 *μ*L of PBS. The assay is based on the interaction of plasma and rabbit red blood cells (HemoStat Laboratories, Dixon, CA) which results in agglutination and natural antibody mediated complement activation. Agglutination scores (HA) represent the interaction between natural antibodies (NAbs) in the plasma and antigens present in the rabbit blood, while lysis scores (HL) reflect the interaction of complement activity and NAbs. Titers for agglutination and lysis were blindly scored from digitized images and correspond to the negative log_2_ of the last plasma dilution at which agglutination or lysis occurred. We assigned half scores to wells that showed intermediate agglutination or lysis.

#### Haptoglobin assay

Haptoglobin concentrations (Hp; mg/mL) present in the plasma were quantified using the manufacturer's instructions provided with the commercially available colorimetric assay (PHASE Haptoglobin assay, Tridelta Development Ltd). Absorbance for all plates was recorded at 630 nm using a Molecular Devices VersaMax Tunable Microplate Reader. We additionally performed a prescan at 630 nm which allowed us to correct for differences in plasma cloudiness and plasma color (Matson et al. 2012).

#### Nitric oxide assay

To quantify the concentrations of nitric oxide (NOx; mmol/L), we used a spectrophotometric assay based on the reduction of nitrate to nitrite by copper‐coated cadmium granules (Sild and Horak 2009). The assay consists of three main steps: deproteinization, nitrate reduction, and a Griess reaction. A standard curve and an among‐plate standard were run in duplicate in each plate. Absorbance was recorded at 542 nm using a Molecular Devices VersaMax Tunable Microplate Reader.

### Statistical analysis

All mixed models were constructed using the lmer function or the glmer function imbedded in the package lme4 in R (Bates et al. 2013) and analyzed using the backward elimination procedure for model reduction. The decision to keep parameters in the model, was based on a significance level of 5%. Model assumptions were checked using Q–Q plots and Shapiro–Wilk normality tests. All statistical analyses were performed in R 2.15.3 (R development core team 2013‐03‐01 release; www.r-project.org). In the case of Hp data, we excluded three outliers from further analysis as they deviated from the mean plus or minus three times the standard deviation plus the average.

#### Weight and tarsus length

To assess whether there were differences in body weight or tarsus length between birds (*N* = 447) who recruited (*N* = 36, *N*_nest_ = 26) and birds who failed to recruit (*N* = 411, *N*_nest_ = 101), we aimed to construct one generalized linear mixed model containing all weight and tarsus measurements as well as all immune measures for each individual and the analysing time (year the sample was analyzed being 2012, 2013, 2014) of each sample. However, due to the fact that weight, tarsus length and field site and analysing time were correlated, we had to divide the model into two separate generalized linear mixed models. The first model contained recruited (yes or no, binomial distribution) as dependent variable, weight and all immune measures (HA, HL, Hp, and NOx) as covariates, field site and nest as random factors with nest being nested in field site. The second generalized linear mixed model contained recruited (yes or no, binomial distribution) as a dependent variable, tarsus and all immune measures (HA, HL, Hp, and NOx) as covariates, field site and nest as random factors with nest being nested in field site.

#### Innate immunity as a predictor of local recruitment

To investigate whether the selected innate immune parameters were suitable predictors of local recruitment, we constructed four separate generalized linear mixed models (one for each immune parameter). Recruitment (yes or no, binomial distribution) was the dependent variable, innate immune parameters (HA, HL, Hp, or NOx) were included as predictor variables, weight was included as a covariate, sample analyzing time (year when lab analysis was conducted), nest and field site were included as random factors, with nest nested in field site. We repeated this approach for data containing only birds that eventually bred. Due to plasma limitations and outliers, sample sizes vary between immune parameters (HA, HL: *N*_recruits_ = 36, *N*_non‐recruits_ = 119, *N*_nests_ = 26; Hp: *N*_recruits_ = 33, *N*_non‐recruits_ = 87, *N*_nests_ = 26; NOx: *N*_recruits_ = 30, *N*_non‐recruits_ = 90, *N*_nests_ = 26).

#### Consistency of innate immune parameters

To investigate whether there is consistency in innate immunity between nestlings and the same individuals as adults, we constructed four separate linear mixed models containing either HA, HL, Hp, or NOx as a dependent variable, age (nestling or adult) as a fixed factor and nest and individual as random factors with individual nested in nest.

To assess whether innate immune parameters of an individual as nestling affect the innate immune parameters of that same individual as an adult, we constructed four linear mixed models. In these models adult immune parameters (HA, HL, Hp, or NOx) were used as dependent variable, time of sampling of the adult blood (winter or breeding season) was included as a fixed factor while nestling immunity was included as a covariate and nest as a random factor. Data for NOx were log10 transformed to meet model assumptions. Due to plasma limitations and outliers, sample sizes vary between immune parameters (HA, HL: *N* = 35; Hp: *N* = 32; NOx: *N* = 18).

To assess the consistency between innate immune parameters of individuals sampled as nestlings and the same individuals sampled as adults, we conducted repeatability estimations using the rptR package in R (Nakagawa and Schielzeth 2010). Data for Hp were square root transformed while NOx data were log10 transformed.

## Results

### Weight and tarsus length

**Figure 2:**
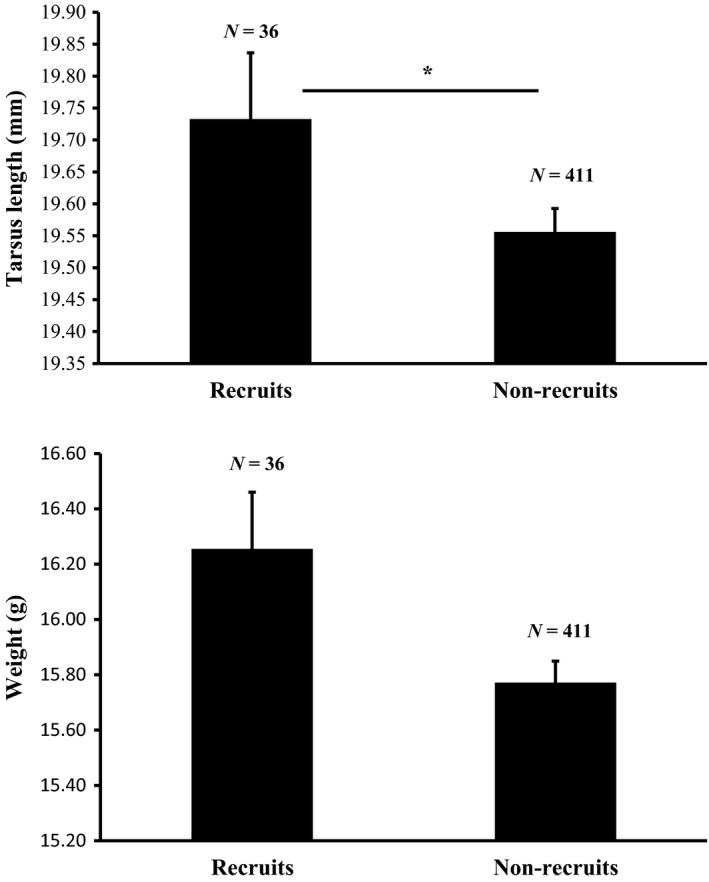
Mean tarsus length ± SE and mean weight ± SE of nestlings who managed to recruit (*N* = 36) and nonrecruited nestlings (*N* = 411). *P ≤ 0.05.

There was a trend for a difference in body weight (*χ*² = 3.56, df = 1, *P *=* *0.06) and a significant difference in tarsus length (*χ*² = 4.75, df = 1, *P *=* *0.03) between birds who recruited and birds who failed to recruit. Birds with a longer tarsus (*β *= 0.70) had significantly more chances to recruit while heavier birds tended to have more chance to recruit (Fig. 2).

### Innate immunity as a predictor of local recruitment

**Figure 3:**
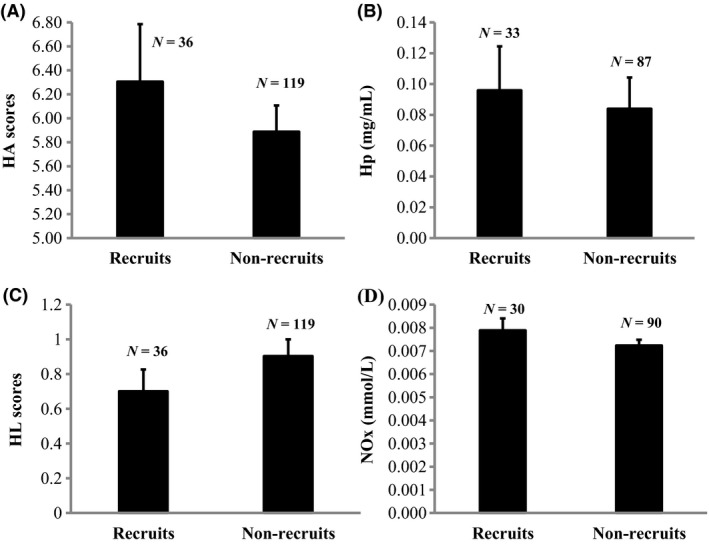
Mean agglutination scores (HA) ± SE (A), haptoglobin concentrations (Hp) ± SE (B), lysis scores (HL) ± SE (C) and nitric oxide concentrations (NOx) ± SE (D) for nestlings who managed to recruit and their nonrecruited siblings.

None of the four innate immune parameters we analyzed in this manuscript were suitable predictors of local recruitment (HA: *χ*² = 0.26, df = 1, *P *=* *0.61; HL: *χ*² = 0.01, df = 1, *P *=* *0.92; Hp: *χ*² = 0.39, df = 1, *P *=* *0.53; NOx: *χ*² = 0.12, df = 1, *P *=* *0.73, Fig. 3).

When we analyzed only the data of birds that eventually bred, patterns still show that the selected innate immune parameters were not suitable predictors of local recruitment (HA: *χ*² = 0.21, df = 1, *P *=* *0.65; HL: *χ*² = 0.02, df = 1, *P *=* *0.88; Hp: *χ*² = 0.45, df = 1, *P *=* *0.50; NOx: *χ*² = 0.16, df = 1, *P *=* *0.71).

### Consistency of innate immune parameters

**Figure 4:**
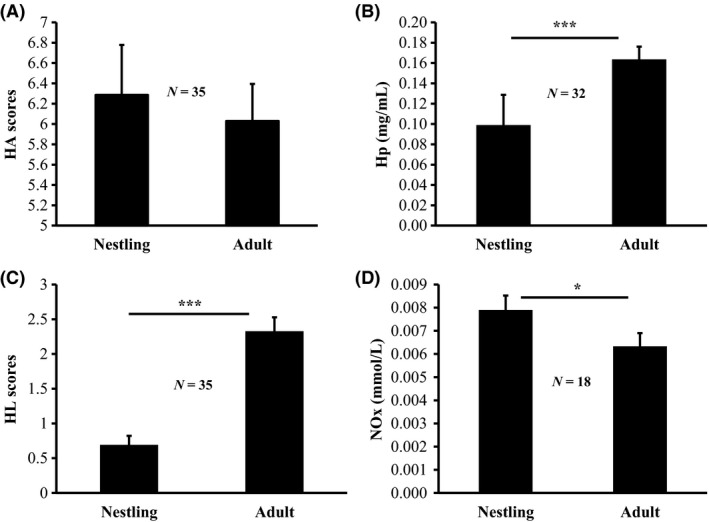
Mean agglutination scores (HA) ± SE (A), haptoglobin scores (Hp) ± SE (B), lysis scores (HL) ± SE (C) and nitric oxide concentrations (NOx) ± SE (D) for nestlings and the same nestlings as adults. *P ≤ 0.05; ***P < 0.001.

There were significant differences between birds sampled as a nestling versus birds sampled as an adult for three of the four innate immune measures (HA: *χ*² = 0.26, df = 1, *P *=* *0.61; HL: *χ*² = 39.45, df = 1, *P *<* *0.0001; Hp: *χ*² = 16.01, df = 1, *P *<* *0.0001; NOx: *χ*² = 5.84, df = 1, *P *=* *0.02, Fig. 4). Nestlings had lower lysis scores (HL) and lower haptoglobin concentrations than adults, while they had higher nitric oxide concentrations than adult birds (Fig. 4).

**Figure 5:**
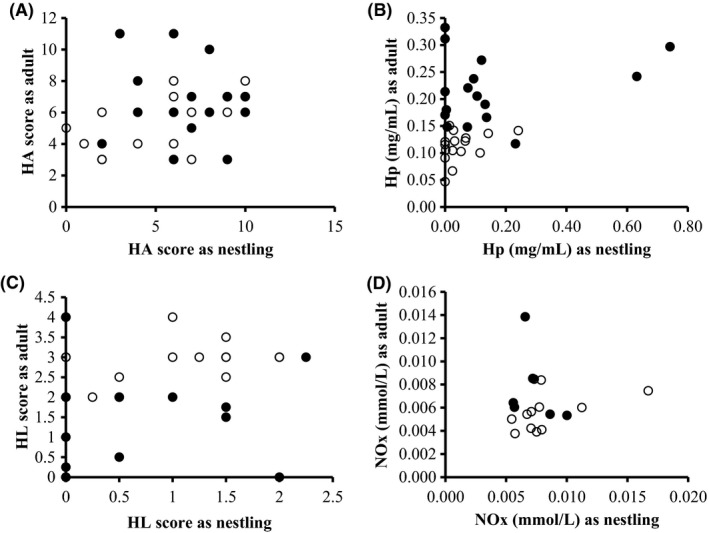
Relationship between innate immune parameters as nestlings and the innate immune parameters of the same individual as adult with (A) agglutination scores HA, (B) haptoglobin concentrations Hp, (C) lysis scores HL, and (D) nitric oxide concentrations NOx. Full circles represent samples of adult birds collected during the breeding season, while open circles represent samples collected during night checks. All data presented here are raw data, whereas for statistics we used transformed data when necessary.

Adult innate immunity was not dependent on nestling innate immune parameters (HA: *χ*² = 1.73, df = 1, *P *=* *0.19; HL: *χ*² = 0.02, df = 1, *P *=* *0.88; Hp: *χ*² = 2.08, df = 1, *P *=* *0.15; NOx: *χ*² = 0, df = 1, *P *=* *1). For HL and NOx, adult birds sampled during winter night checks had higher immune values compared to adults in the breeding season (HL: *χ*² = 15.36, df = 1, *P *<* *0.0001; NOx: *χ*² = 32.75, df = 1, *P *<* *0.001, Fig. 5). For Hp, the opposite was true, with adults sampled during the breeding season having higher haptoglobin levels than adult birds sampled in winter (Hp: *χ*² = 26.45, df = 1, *P *<* *0.001, Fig. 5). For HA there was no effect of sampling time (HA: *χ*² = 1.74, df = 1, *P *=* *0.19, Fig. 5).

Neither of the innate immune parameters was repeatable across life stages, that is birds sampled as nestlings and as adults, although there were indications of a trend for HA (HA: *R *=* *0.28, *P *=* *0.06; HL: *R *=* *0, *P *=* *1; Hp: *R *=* *0, *P *=* *1; NOx: *R *=* *0, *P *=* *1).

## Discussion

We examined whether innate immune parameters can predict local recruitment and additionally we explored within individual consistency of innate immune parameters in wild great tits. We found that size but not immunity measured in terms of natural antibodies, activity of the complement system, haptoglobin concentrations or nitric oxide concentrations affected the likelihood of local recruitment. Innate immunity was, furthermore, not consistent within individuals across life history stages.

### Weight and tarsus length

In birds, body mass and structural size (estimated via tarsus length) are the variables that are most frequently positively associated with postfledging survival (Tinbergen and Boerlijst 1990; Both et al. 1999; Naef‐Daenzer et al. 2001; Perrins and McCleery 2001; Monrós et al. 2002; Brotons and Broggi 2003; Lopez‐Rull et al. 2011; Losdat et al. 2013). In line with these previous studies, we found a trend for a difference in body weight and a significant difference in tarsus length between birds who managed to recruit and birds who failed to recruit, indicating that mass and size at fledging can indeed facilitate local recruitment (Fig. 2). Thus, despite restrictions due to comparatively low recruitment rates of 3.7% compared to above 5% in other years in this (pers. observation) or other great tit populations (e.g., Naef‐Daenzer et al. 2001; Monrós et al. 2002; Losdat et al. 2013), we have sufficient statistical power to establish this relationship, which is relevant in order to scale all subsequent results.

### Innate immunity as a predictor of local recruitment

We found no evidence that nestling natural antibodies, nestling complement activity, nestling haptoglobin concentrations or nestling nitric oxide concentrations facilitated local recruitment (Fig. 3). Even when we analyzed only data of birds that eventually bred, patterns still show that the selected innate immune parameters were not suitable predictors of local recruitment. Our results contrast with the findings of a number of previous studies focusing on cell‐mediated immunity (PHA response), which reported positive associations between their immune measure and recruitment (Møller and Saino 2004; Cichon and Dubiec 2005; Moreno et al. 2005; Lopez‐Rull et al. 2011; Bowers et al. 2014). However, the cell‐mediated immunity as measured via a PHA challenge has repeatedly been shown to be condition dependent (Alonso‐Alvarez and Tella 2001; Thompson et al. 2014). Positive associations of cell‐mediated immunity with survival are, therefore, potentially confounded by the fact that survivors are generally heavier (Alonso‐Alvarez and Tella 2001). However, based on the limited evidence so far, innate immunity appears to be less condition dependent indicating that the different axes of the immune system may be affected in different ways (Thompson et al. 2014). This may, together with the results of this study, suggest that it is not immunity per se that confers a long‐term survival benefit (but see Lobato et al. 2005; Cichon and Dubiec 2005). However, the power of our study is still rather low due to the relatively low number of recruits we retrieved compared to other studies (Naef‐Daenzer et al. 2001; Monrós et al. 2002; Losdat et al. 2013), and we do not want, therefore, to speculate too much. Furthermore, besides the innate immune parameters we selected, there are obviously many other innate immune components that might facilitate recruitment and which could not be further explored due to limited sample volume. Our conclusions are, therefore, restricted to the parameters of the innate immune system that we investigated.

### Consistency of innate immune parameters

Young animals rely heavily on their innate immunity since they still have very poor specific immune responses (Klasing and Leshchinsky 1999). However, we show that the innate immune system may not be fully developed either. HL and Hp scores were both higher in adults compared to nestlings (Fig. 4). The fact that HL and Hp scores are higher in adults may indicate that maturation of these traits leads to an improved immune function (Palacios et al. 2009). There is, indeed, evidence for differences in innate immunity between nestlings and adult tree swallow (*Tachycineta bicolor*), with adults having higher innate immune responses, measured as the ability of whole blood to kill *Escherichia coli*, compared to nestlings (Stambaugh et al. 2011). Other evidence comes from Palacios et al. (2009), who showed that adult tree swallows had higher natural antibody titers and higher complement‐mediated lysis titers compared to nestlings. NOx concentrations in contrast were higher in nestlings compared to adults (Fig. 4). The higher NOx levels observed in nestlings may indicate that this immune trait plays an important role at an early age, and that it might compensate for other traits of the immune system that have not yet fully developed (e.g., traits of the humoral immune system).

Thus, immunity is plastic and shows a developmental process when individuals grow (Ardia et al. 2011; Ygberg and Nilsson 2012). But, one could expect that there may be within individual consistency due to heritability or priming effects (Cichon et al. 2006; Yang et al. 2009). In fact, there should be genetic variation in immunity in order for immunity to evolve and to respond to selection. This may in particular apply to the innate immune system as most of the information built up by the memory function of the acquired immune functions is lost at death. Thus, nestlings with high innate immune function may also have a high immune function as adults. However, most studies mentioned above focused on the comparison between nestlings and adults in general and not on the within individual level (but see Love et al. 2008). To the best of our knowledge, information on within individual consistency in innate immunity is generally lacking. Based on our results, there is no evidence for within individual consistency for the selected innate immune measures, which was rather unexpected. A possible explanation may be that the immune system is strongly shaped by external environmental conditions (Lifjeld et al. 2002; Garvin et al. 2006; Pitala et al. 2007; Hegemann et al. 2012), as also indicated by the difference in innate immunity with time of the year. Thus there is probably a strong environmental component which is rather unpredictable, and it may require larger sample sizes.

## Conclusions

Size appeared to be more relevant for recruitment than innate immunity, which may indicate that the causes of postfledging mortality may relate to predation, starvation, and competition rather than infectious diseases. Innate immunity was present at the nestling stage, where it probably fulfils a significant role in early immune defenses. It nevertheless significantly changed when individuals grew from nestlings to adults, which probably relates to maturation as well as the increased availability of other (adaptive) immune traits. Innate immunity was not consistent throughout an individual's life at least for the immune parameters we measured. This is a highly relevant finding for a broad range of behavioral and evolutionary ecological topics, but currently only very limited evidence on within‐individual consistency in immune traits exists. We, therefore, encourage further exploration of within‐individual variation of innate immunity and preferentially in combination with measures of adaptive immunity. This is necessary to ultimately understand the complex immune system and its role in recruitment.

## Conflict of Interest

None declared.
